# Effect of pain on depression among nursing home residents: Serial mediation of perceived social support and self‐rated health. A cross‐sectional study

**DOI:** 10.1111/ggi.14067

**Published:** 2020-10-22

**Authors:** Ye Chen, Meiliyang Wu, Tieying Zeng, Chaohua Peng, Meizhen Zhao, Qi Xiao, Mengmei Yuan, Ke Zhang, Xuejun Wang

**Affiliations:** ^1^ Department of Nursing, Tongji Hospital, Tongji Medical College Huazhong University of Science and Technology Wuhan China; ^2^ School of Nursing, Tongji Medical College Huazhong University of Science and Technology Wuhan China

**Keywords:** depression, pain, perceived social support, self‐rated health, serial mediation

## Abstract

**Aim:**

The aim of this study was to investigate how perceived social support and self‐rated health together could mediate the relationship between pain and depression among Chinese nursing home residents with pain.

**Methods:**

The study was conducted in 38 nursing homes in 13 cities in China. A convenience sample of 2154 older adults responded to the questionnaire survey. A mediation analysis was performed on the data of 990 participants with pain. The data were collected by a questionnaire consisting of socio‐economic and demographic characteristics, the Geriatric Depression Scale‐15, the Multidimensional Scale of Perceived Social Support, Self‐rated Health Scale and the Numerical Rating Scale of pain. The sample was subdivided by sex. Descriptive analysis, *t*‐tests, chi‐squared tests, Mann–Whitney *U*‐tests, Spearman correlation analyses and the bootstrap method were used to analyze data.

**Results:**

The prevalence rate of pain and depression among nursing home residents were 46.0% and 20.7% respectively. Pain, perceived social support and self‐rated health were all significantly correlated with depression (*r* = 0.217, *P* < 0.01; *r* = −0.216, *P* < 0.01; *r* = 0.385, *P* < 0.01, respectively). Perceived social support and self‐rated health independently and in series partly explained the relationship between pain and depression.

**Conclusions:**

The results of the study showed that pain was associated with low perceived social support first, and then poor self‐rated health, which was in turn related to the development of depression among nursing home residents with pain. For nursing home residents, perceived social support and self‐rated health as an internal resource can affect the ability to overcome the suffering of pain and reduce the level of depression. **Geriatr Gerontol Int 2020; 20: 1234–1240**.

## Introduction

Pain is a common complaint in older adults who live in long‐term care facilities. The prevalence of pain among nursing home residents varies from 27.8% to 79.5%,^1^ influenced by the research methods and data sources. Unrelieved pain is associated with impaired physical functioning (impaired mobility, falls), and mental and social functioning (depression, anxiety, social withdrawal).^2^

Comorbidity of pain and depression has long been recognized and is associated with a greater burden to the individual and society than either condition alone. It has been estimated that 30–60% of patients in pain report comorbid depression.^3^ Although the causal direction between pain and depression is still unclear, a longitudinal study found that pain was a risk factor for developing depression after excluding participants with a history of depression.^4^ Pain can significantly affect people's activities of daily living and increase psychological distress, leading to mood disorders such as depression.^5^ A study has documented that a one‐point increase in pain was associated with a 0.48‐point increase in depression in patients with dementia using mixed model analyses.^6^

The factors and underlying mechanisms linking pain with depression are complicated, including neurobiology, environmental factors and psychosocial factors.^3^ The cognitive‐behavioral mediation model^7^ suggests the pain may contribute to a negative bias in perceptions of health or personal mastery. The negative bias may predispose a person for emotional distress such as depression. Previous studies have explained that depression was associated with poorer self‐rated health and perceived low social support.^8^, ^9^ Accordingly, pain alone may be not a sufficient condition for the development of depression and some cognitive appraisal variables may mediate this relationship. Cognitive factors have become an avenue of research into the question of why some people with pain develop depression symptoms while others do not.^7^ Finding mediators involved in the relationship between pain and depression may be important for subsequent relevant interventions for older adults with pain to reduce the development of depression secondary to pain.

### *Mediation effect*

Perceived social support (PSS), which is defined as the individual cognitive appraisal about the availability of varied types of support from network associates, has often been linked to physical and mental health.^10^ Pain may contribute to a tendency of pain catastrophizing, which can produce maladaptive changes in people's social support.^11^ A qualitative study indicated that people who experienced pain reported weakened social ties and a tendency towards social isolation compared with the situation before they had pain.^12^ Based upon these previous findings, we hypothesize that experiencing pain may contribute to a feeling of low social support. Moreover, it has been proved that low PSS is significantly correlated to higher levels of depression.^9^ Therefore, in the current study, we hypothesize that PSS might be the potential mediating variable between pain and depression.

People's self‐rated health (SRH) in old age refers to the cognitive appraisal of their own health status, which in turn is related to nursing home placement, mortality and emotional well‐being.^13^ SRH is an active cognitive process that is not guided by formal, agreed rules or definitions. It is probably the most informative measure of health status as it is a more inclusive measure than other direct health indicators used in population studies.^14^ It has been proved that pain has an adverse impact on SRH in older adults.^15^ Moreover, Mulsant *et al*.^16^ found that SRH is strongly and independently associated with depressive symptoms even after controlling for physical illness and functional disability. Therefore, we hypothesize that SRH may mediate the relationship between pain and depression. In addition, pain was shown to have a negative role on PSS,^12^ PSS was in turn found to have a negative impact on SRH.^17^ With these findings in mind, we also hypothesize that pain is sequentially associated with low PSS first and then poor SRH, which is in turn related to depression.

### *Hypothesized model*

**Figure 1:**
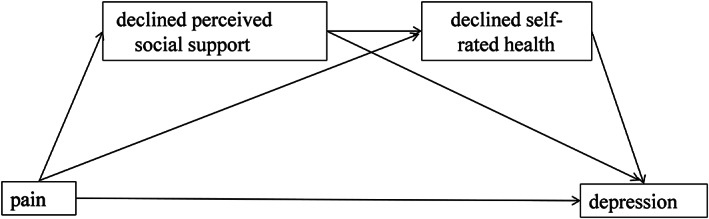
Relationships among pain, perceived social support, self‐rated health and depression.

Although the negative impact of pain on depression has already been examined,^6^ the mechanisms underlying the association between pain and depression have yet to be elucidated. Furthermore, we believe that to understand these relations fully, it is important to determine the roles PSS and SRH have on depression. According to the cognitive‐behavioral model^7^ and previous findings,^8^, ^15^, ^16^, ^17^ we assume that the relationship between pain and depression can be mediated by PSS and SRH independently and in series. The hypothesized model is shown in Figure 1. The model assessed three indirect associations: (i) association between pain and depression, indirectly via PSS; (ii) association between pain and depression, indirectly via SRH; (iii) association between pain and depression, indirectly via PSS and SRH in series. The model also assessed the direct association between pain and depression while controlling for all mediators included in the model.

Sex differences have been found in pain, social support, SRH and depression across the aging process.^17^ To this end the aims of the present study were two‐fold, i.e., (i) to test a serial‐multiple mediation model among pain, PSS, SRH and depression in nursing home residents with pain, and (ii) to explore potential sex differences in the hypothesized model.

## Methods

### *Study design and participants*

We conducted a cross‐sectional survey based on a convenience sample of nursing home residents in 13 cities (Wuhan, Yichang, Huanggang, Hangzhou, Jinhua, Haikou, Sanya, Xi'an, Xianyang, Shenzhen, Beijing, Chengdu, Chongqing) in China, from September 2017 to June 2018. Participants were recruited via word‐of‐mouth advertising. In total, 2171 residents volunteered, met the inclusion criteria, and were recruited from 38 nursing homes; 17 participants withdrew their consent to participate during the interview (effective response rate was 99.2%). The inclusion criteria were: (i) aged ≥60 years, (ii) able to communicate in Mandarin, and (iii) willing to participate in the interview. The exclusion criteria were (i) cognitive impairment, and (ii) severe hearing impairment (except using hearing amplifiers). The questionnaire was collected through personal face to face interviews. Six interviewers who were all postgraduates from our hospital conducted the interviews. Before the survey, a training program was carried out to reduce interviewer variability effect on information gathering.

### *Ethical considerations*

The study was approved by the Ethics Committee of Tongji Hospital, Tongji Medical College, and Huazhong University of Science and technology. Permission from the administrations of the nursing homes was secured and a confidentiality agreement was signed. Informed consents were obtained from all respondents. They were informed that their anonymity would be guaranteed and the results obtained would be used only for this research. Complete confidentiality of the data related to older people was guaranteed.

### *Measurements*

#### *Socio‐economic and demographic characteristics*

A self‐designed questionnaire was used to collect information on age, sex, marital status, educational level, perceived financial stress and independence in self‐rated activities of daily living (ADL).

#### *Short Portable Mental Status Questionnaire*

The Short Portable Mental Status Questionnaire (SPMSQ) is a brief cognitive screening instrument, which includes 10‐item testing orientation to time and place, memory, current event information and calculation. The total number of errors is computed and it ranges from 0 to 10. A cut‐off point of five or more errors suggesting patients with cognitive impairment was used.^18^ When the respondent got five or more incorrect answers, he/she would be excluded from the study.

#### *Self‐rated health*

SRH is a reliable, quick and widely used assessment for population health monitoring in epidemiological and clinical studies.^13^ It was assessed using a single item about the general health from the SF‐36. Participants were asked，“How would you rate your overall health at the present time?” and the choice is scored by a Likert‐type scale ranging from 1 (very good) to 5 (poor).

#### *Pain*

Pain was defined as any pain experienced in the preceding week. We applied the Numerical Rating Scale (NRS) of pain, which has been proven to be valid and acceptable in older people,^19^ to assess the pain intensity. Participants were required to rate their pain intensity from 0 to 10, with 0 representing no pain and 10 representing the worst imaginable pain. A score of ≥1 is considered to indicate pain.

#### *Depression*

Depression was assessed with the Geriatric Depression Scale‐15 (GDS‐15). The 15‐item questionnaire is a valid and convenient tool, which has been frequently used to screen depression in older adults.^9^ Participants were asked to describe their feelings in the previous week. The answer “no” in items 1, 5, 7, 11 and 13 and the answer “yes” in the other questions suggest depression. Each item was given one point if the answer indicated depression. Scores range from 0 to 15, with a higher score indicating a higher level of depression. A score of ≥5 indicates the presence of clinically significant symptoms of depression. The internal consistency index calculated on our data (Cronbach's alpha) was 0.81.

#### *Perceived social support*

We assessed the PSS with the Multidimensional Scale of Perceived Social Support (MSPSS). The MSPSS consists of 12 items referred to as three sources of support: family, friends and significant other.^20^ Each factor group includes four items and each item is scored by a Likert‐type scale ranging from 1 (very strong disagree) to 7 (very strong agree). Therefore, the total score ranges between 12 and 84. The high scores indicate high PSS. The internal consistency index (Cronbach's alpha) calculated on our data was 0.92.

### *Statistical analyses*

All analyses were stratified by sex. Differences in characteristics between sex were examined with descriptive analyses. The level of statistical significance was set at *P* < 0.05. Means of continuous (normal distribution) and the distribution of categorical variables were compared using *t*‐tests and chi‐squared tests respectively. Non‐parametric Mann–Whitney *U*‐tests were computed to compare non‐normally distributed data between women and men. Spearman correlation analyses of the four key variables (pain, PSS, SRH, depression) were performed using IBM SPSS Statistics version 21.0 (IBM Corp., Armonk, NY, USA). Mediation analyses were conducted in residents who reported pain with the Mplus 7.4 (Muthén and Muthén, 1998–2015) software to calculate the mediation parameters for the total, direct and indirect effects, using a serial mediation model with pain as a predictor, two serial mediators (PSS → SRH), and depression as the outcome. Bootstrapping, a non‐parametric resampling technique was used, as it is more robust than the traditional Baron and Kenny approach or Sobel's test for testing mediation without the requirement of distributional assumptions. Bootstrapping can be used for making inferences about indirect effects in an intervening variable model, regardless of the complexity and number of the paths between predictors and outcomes.^21^ Bias‐corrected 95% confidence interval was used to assess the significance of direct and indirect effects. If the 95% confidence interval did not encompass zero, the effect would be considered significant. Covariates including age, marital status, education level, perceived financial stress and ADL were statistically accounted for. All analyses were two‐tailed.

## Results

### *Sample characteristics*

Of the 2154 participants, 1382 were women and 772 were men. The mean ± SD age was 82.0 ± 7.0 years. The majority of the participants were single (65.2%) and reported no financial stress (82.4%). In total, 40.3% of the participants were independent, 52.1% were partially dependent and 7.5% were dependent. The mean ± SD scores were 3.4 ± 0.9 and 60.1 ± 15.2 in SRH and PSS respectively. In total, 46.0% of the participants reported mild to severe pain. With regard to depression, 20.7% of the participants had depressive symptoms.

A detailed overview of sex differences is provided in Table 1. There was no significant difference in SRH scores between women and men. The PSS score was significantly higher in women than in men (*P* < 0.01). In total, 52.0% of the women versus 35.2% of the men had pain, with the difference being statistically significant (*P* < 0.01). Men also had a higher level of depression than women did (*P* < 0.01).

**Table 1:** Characteristics of the study sample distributed by gender

Variable^†^	Men	Women	Total	*t*/z/χ^2^	*P*
Age (years)	81.9 ± 8.3	82.1 ± 6.2	82.0 ± 7.0	−0.673	0.501
Marital status, *n* (%)				122.186	**<0.001**
Single^‡^	386 (50.0)	1018 (73.7)	1404 (65.2)		
Married	386 (50.0)	364 (26.3)	750 (34.8)		
Education, *n* (%)				30.431	**<0.001**
Primary school or below	240 (31.2)	564 (41.0)	804 (37.5)		
Middle and high school	330 (42.9)	572 (41.6)	902 (42.0)		
College/university or above	200 (26.0)	240 (17.4)	440 (20.5)		
ADL, *n* (%)				7.539	**0.023**
Independence	320 (41.5)	549 (39.7)	869 (40.3)		
Partial dependence	380 (49.2)	743 (53.8)	1123 (52.1)		
Dependence	72 (9.3)	90 (6.5)	162 (7.5)		
Perceived financial stress, *n* (%)				5.179	0.159
None	638 (82.6)	1136 (82.2)	1774 (82.4)		
Mild	90 (11.7)	164 (11.9)	254 (11.8)		
Moderate	30 (3.9)	70 (5.1)	100 (4.6)		
Severe	14 (1.8)	12 (0.9)	26(1.2)		
Self‐rated health	3.5 ± 1.0	3.4 ± 0.9	3.4 ± 0.9	1.514	0.13
Pain (NRS)					
0	500 (64.8)	664 (48.0)	1164 (54.0)	55.755	**<0.001**
≥1	272 (35.2)	718 (52.0)	990 (46.0)		
Perceived social support (MSPSS)	58.5 ± 15.5	61.0 ± 14.9	60.1 ± 15.2	−3.018	**0.003**
Family	22.4 ± 5.5	22.6 ± 5.2	22.5 ± 5.3	−0.006	0.995
Friend	14.1 ± 7.4	15.9 ± 7.4	15.3 ± 7.5	−4.984	**<0.001**
Significant other	22.0 ± 5.6	22.5 ± 5.4	22.3 ± 5.5	−2.061	**0.039**
Depression (GDS‐15)	2 (1.4)	2 (0.4)	2 (0.4)	−2.659	**0.008**
<5	596 (77.2)	1112 (80.5)	1708 (79.3)	3.013	0.083
≥5	176 (22.8)	270 (19.5)	446 (20.7)		

^†^ There are some missing values, the number of people included for analysis is in the table.

^‡^ Including single, widowed, divorced and separated.

NRS, Numerical Rating Scale (NRS) of pain; MSPSS, the Multidimensional Scale of Perceived Social Support; GDS‐15, the Geriatric Depression Scale‐15.

Values are presented as mean ± SD, median (IQR) and *n* (%).

The bold values mentioned in the table reached statistally significance, *P* < 0.05.

### *Correlations*

In the combined‐sex sample, the Spearman rank correlation analyses showed that SRH score was positively correlated with depression (*r* = 0.385, *P* < 0.01) and pain (*r* = 0.191, *P* < 0.01), and PSS was negatively correlated with depression (*r* = −0.216, *P* < 0.01) and pain (*r* = −0.052, *P* < 0.05). It meant that pain and depression might be frequently accompanied by poor SRH and low PSS among nursing home residents. Depression, as an independent variable, was also found to be positively correlated with pain (*r* = 0.217, *P* < 0.01). The correlations between the four variables in men and women are displayed in Table 2. The same relations were found in women and men except that the correlation between pain and PSS was not significant in men.

**Table 2:** Sex‐specific correlations between pai, self‐rated health, perceived social support and depression (men, *n* = 772; women, *n* = 1382)

Variable	Pain	Self‐rated health	Perceived social support	Depression
Pain	–	0.195**	−0.076*	0.235**
Self‐rated health	0.211**	–	−0.175**	0.401**
Perceived social support	−0.056	−0.107*	–	−0.264**
Depression	0.219**	0.364**	−0.256**	–

*P < 0.05, **P < 0.01. Values above the diagonal refer to women subgroup, values below the diagonal refer to men subgroup.

### *Serial mediation analysis*

**Figure 2:**
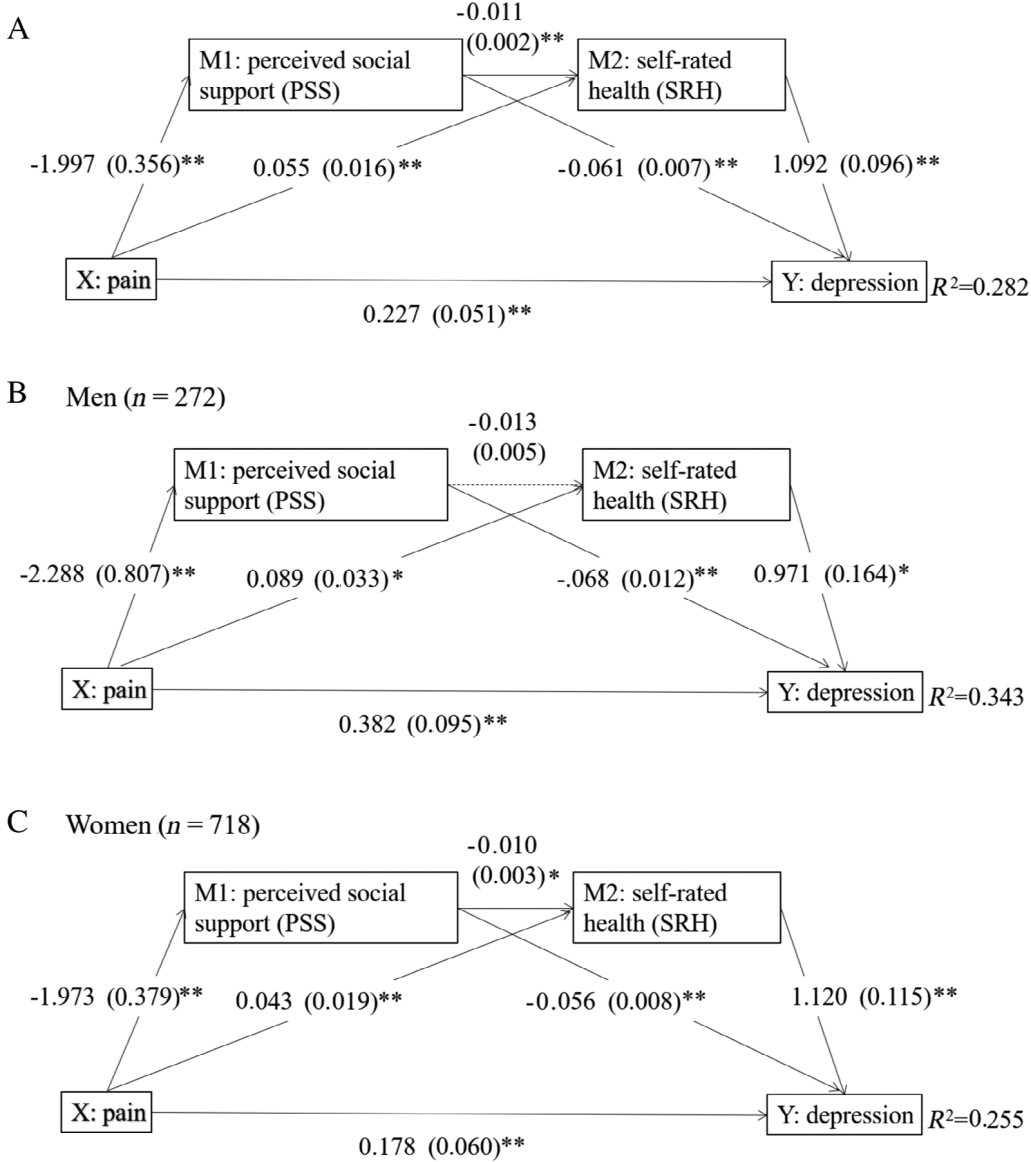
Combined sex (a) and gender‐specific (b, c) serial mediation models. Values are the unstandardized regression coefficient (bootstrap standard error). Pathways highlighted by dotted line did not reach significance, *P* > 0.05. **P < 0.01. *P < 0.05.

To test the hypothesized model, the direct, indirect and total effects of pain on depression were examined with PSS and SRH as potential mediating variables. In combined‐sex models (Table 3; Fig. 2a), we saw that pain was positively associated with depression through three indirect pathways: (i) through PSS solely (indirect effect = 0.122, *P* < 0.01); (ii) through SRH solely (indirect effect = 0.060, *P* < 0.01); and (iii) through PSS and SRH in series (indirect effect = 0.024, *P* < 0.01). Therefore, the greater the pain was, the more it would contribute to a negatively biased self‐perception of social support and health, which might sequentially lead to depression. The full model accounted for 28.2% of the variance in depression.

**Table 3:** Analysis of the total, direct and indirect effects in the sample

				Bootstrapping (5000)			
		Product of coefficients		Percentile 95% CI		Bias corrected percentile 95% CI	
Point estimate		SE	*Z*	Lower	Upper	Lower	Upper
Combined model (*n* = 990)							
Total	0.433**	0.056	7.675	0.322	0.543	0.361	0.601
Direct	0.227**	0.051	4.429	0.127	0.328	0.170	0.390
Indirect	0.206**	0.032	6.462	0.143	0.268	0.148	0.261
X → M1 → Y	0.122**	0.024	5.026	0.074	0.169	0.075	0.149
X → M2 → Y	0.060**	0.018	3.388	0.025	0.094	0.037	0.125
X → M1 → M2 → Y	0.024**	0.007	3.362	0.010	0.038	0.002	0.029
Men (*n* = 272)							
Total	0.652**	0.118	5.520	0.421	0.884	0.414	0.928
Direct	0.382**	0.095	4.027	0.196	0.567	0.172	0.629
Indirect	0.271**	0.068	3.962	0.137	0.405	0.157	0.411
X → M1 → Y	0.156**	0.054	2.877	0.050	0.262	0.080	0.260
X → M2 → Y	0.087*	0.034	2.516	0.019	0.154	0.024	0.191
X → M1 → M2 → Y	0.029	0.016	1.732	−0.004	0.061	−0.006	0.055
Women (*n* = 718)							
Total	0.358**	0.065	5.548	0.233	0.487	0.284	0.566
Direct	0.178**	0.060	2.965	0.060	0.295	0.118	0.374
Indirect	0.180**	0.035	5.137	0.113	0.252	0.119	0.242
X → M1 → Y	0.110**	0.022	3.257	0.028	0.113	0.057	0.141
X → M2 → Y	0.048**	0.025	4.104	0.053	0.151	0.022	0.126
X → M1 → M2 → Y	0.022*	0.004	2.158	0.001	0.018	0.006	0.029

*P < 0.05, **P < 0.01. All the values are unstandardized. X1 = pain, M1 = perceived social support, M2 = self‐rated health, Y = depression.

CI, confidence interval.

In agreement with findings in the combined sample, the direct and indirect effects were significant in both men and women (Table 3, Fig. 2b,c). When mediation pathways were assessed in women only (Table 3, Fig. 2c), pain was indirectly associated with depression, through PSS and SRH independently and in series. In the male model (Table 3, Fig. 2b), the indirect association between pain and depression existed through PSS and SRH independently with no mediation through PSS and SRH in series.

## Discussion

In this study, we investigated the prevalence of pain and depression among Chinese nursing home residents, examined a serial mediation model and tested the sex difference of the model. We found that 46.0% of respondents in our study reported mild to severe pain, which was higher than the rate found in long‐stay US nursing home residents (38.6%),^22^ and considerably higher than the rates reported in Chinese community residents aged ≥60 years (33%).^23^ The result suggested that pain poses a great challenge to Chinese nursing home residents. In addition, 20.7% of respondents had symptoms of depression in our study, which was similar to the result of Tiong *et al*.^24^ In total, 375 residents aged ≥55 years were surveyed in six nursing homes in Singapore and obtained a prevalence rate of 21.1%. However, our result was lower than the finding (26.6%) of Zhao *et al*.,^25^ who investigated a total of 323 Chinese older nursing home residents with a seven‐item Hospital Depression Scale. Sampling structure and measurement tools may have contributed to the inconsistency. Our results suggested a high prevalence rate of pain and depression in nursing homes, thus appropriate intervention is needed. We examined the mediators that could potentially contribute to the development of depression following pain among institutionalized older people. Once the true mediating process is identified, more efficient and powerful interventions can be developed because these interventions can focus on variables in the mediating process.

Consistent with our hypothesis model, pain was closely correlated with depression, and the relationship between pain and depression was mediated by PSS and SRH. There were three specific indirect effects in the serial mediation model. First, PSS worked as a mediator between pain and depression. A possible explanation is that pain may interfere with daily activities,^5^ decrease the frequency of contact with others, and even lead to social withdrawal. These may result in poor PSS and further, accelerate the development of depression.^5^ This finding was partly similar to a previous study, which suggested that social support mediated the pain–depression association in 274 injured workers.^8^ Second, the pain would impact depression when it was severe enough to affect SRH. On the one hand, SRH could be a predictor of the level of pain experienced as a summative subjective indicator of underlying clinical health status.^15^ On the other hand, pain is strongly associated with the perception of losing independence and autonomy.^5^ When people feel obvious changes in their routine, activities and social life due to physical or mental limitations, they will recognize their own decline in comparison with themselves in past time or with their peers.^26^ Therefore, this may lead to a decline in SRH and, in turn, poor SRH was associated with depression.^16^ Third, our study revealed that PSS and SRH in series affected the relationship of pain with depression among nursing home residents. A previous study had shown that pain had a negative impact on PSS.^11^ People with low PSS could have unhealthy behaviors such as having less physical activities and smoking, which might lead to poor SRH.^27^ Finally, poor SRH was related to depression.^16^

When sex‐specific models were conducted, a non‐significant indirect association through PSS and SRH in series was seen in males. This suggested that in our sample, men had low odds of having poor SRH in the absence of social support. The result was in accordance with a study, which indicated social support were significant correlates of women's SRH but carried no such weight among men.^14^ In general, very small differences existed between the sex models. The two models followed the same trend as the one observed for the total sample model.

To our knowledge, no study so far has examined the relationship between pain and depression using a serial mediation model of PSS and SRH among nursing home residents. Our study proves that pain not only has a direct association with depression, but also has indirect associations with depression via the mediating role of PSS and SRH alone or combined in series. The results suggest that more attention should be paid to nursing home residents with pain, who also may feel a low level of social support and SRH, and tend to have a higher level of depression than those who have a higher level of PSS or SRH. Given that PSS and SRH mediate the relationship between pain and depression, intervention measures that contribute to enhance PSS and SRH may be promising for older adults in pain to develop into a less depressive status. We propose several ways to do this: effective interventions such as cognitive‐behavioral therapy, which focuses on restructuring the negative cognition of people into a realistic appraisal,^28^ could be explored to improve PSS and SRH. Meanwhile, assessment and effective management of pain should be provided in gerontological practice to reduce pain and depression.^29^

The strength of our study is the large amount of data from nursing home residents with a variety of socio‐economic backgrounds. In addition, we have found that the relationship between pain and depression can be mediated by PSS and SRH, which may provide a perspective of how pain contributes to the development of depression from the standpoint of psychology.

Several limitations to our study need to be acknowledged. First, considering the language barrier, we did not take the multi‐racial and multi‐cultural provinces such as Sinkiang and Tibet into account. In addition, due to the limited costs and number of interviewers, a convenience sampling method was adopted. These factors limited the representation of the national population and the external validity of the study. Second, limited by the cross‐sectional design, the causal pathways of the results of the mediation analyses should be interpreted with caution. In subsequent research, longitudinal data should be collected to prove the predictive power of the mediation model.

## Disclosure statement

The authors declare no conflict of interest.
